# Case study: autoimmune hepatitis with cirrhosis and pancytopenia since 7 weeks’ gestation

**DOI:** 10.1515/crpm-2024-0007

**Published:** 2024-04-26

**Authors:** Rachel Lee, Zenobia Gonsalves, Sophia Wang, Ayesha Hussain, Kimberly Herrera

**Affiliations:** Department of Obstetrics, Gynecology and Reproductive Medicine, Division of Maternal Fetal Medicine, Stony Brook University Hospital, Stony Brook, NY, USA; Department of Obstetrics and Gynecology, Stony Brook University Hospital, Stony Brook, NY, USA

**Keywords:** severe pancytopenia, autoimmune hepatitis in pregnancy, cirrhosis in pregnancy, spontaneous bacterial peritonitis, hepatic encephalopathy, puerperal sepsis

## Abstract

**Objectives:**

Autoimmune hepatitis (AIH) is a chronic inflammatory disease of unknown etiology and AIH in pregnancy is associated with many adverse maternal and fetal outcomes. The purpose of this report is to share insight into management of AIH-induced pancytopenia unresponsive to steroids and transfusions.

**Case presentation:**

A 29-year-old G4P0121 female with history of spontaneous bacterial peritonitis (SBP) and severe pancytopenia secondary to AIH was found to be incidentally pregnant at 7 weeks gestation. Despite multiple blood transfusions and steroids, her pancytopenia was unresponsive to therapy. At 33 weeks, she underwent primary cesarean section for persistent category II fetal heart tracing and delivered a viable infant. Delivery was complicated by hemorrhage requiring multiple blood products. Postpartum course was complicated by sepsis secondary to urinary tract infection, and decompensated cirrhosis with hepatic encephalopathy and coagulopathy. Both fetus and mother have recovered well 3 months post-delivery.

**Conclusions:**

This case highlights the challenges in management of AIH in pregnancy, particularly the difficulty in treating severe unresponsive pancytopenia as well as balancing the need for immunosuppression with the increased risk of infection that may lead to sequelae such as SBP and puerperal sepsis.

## Introduction

Autoimmune hepatitis (AIH) is a chronic inflammatory disease of unknown etiology characterized by progressive hepatocellular inflammation and necrosis that may progress to cirrhosis. AIH in pregnancy is associated with many adverse maternal and fetal outcomes, including severe pancytopenia unresponsive to therapy which was seen in this case.

## Case presentation

**Figure 1:**
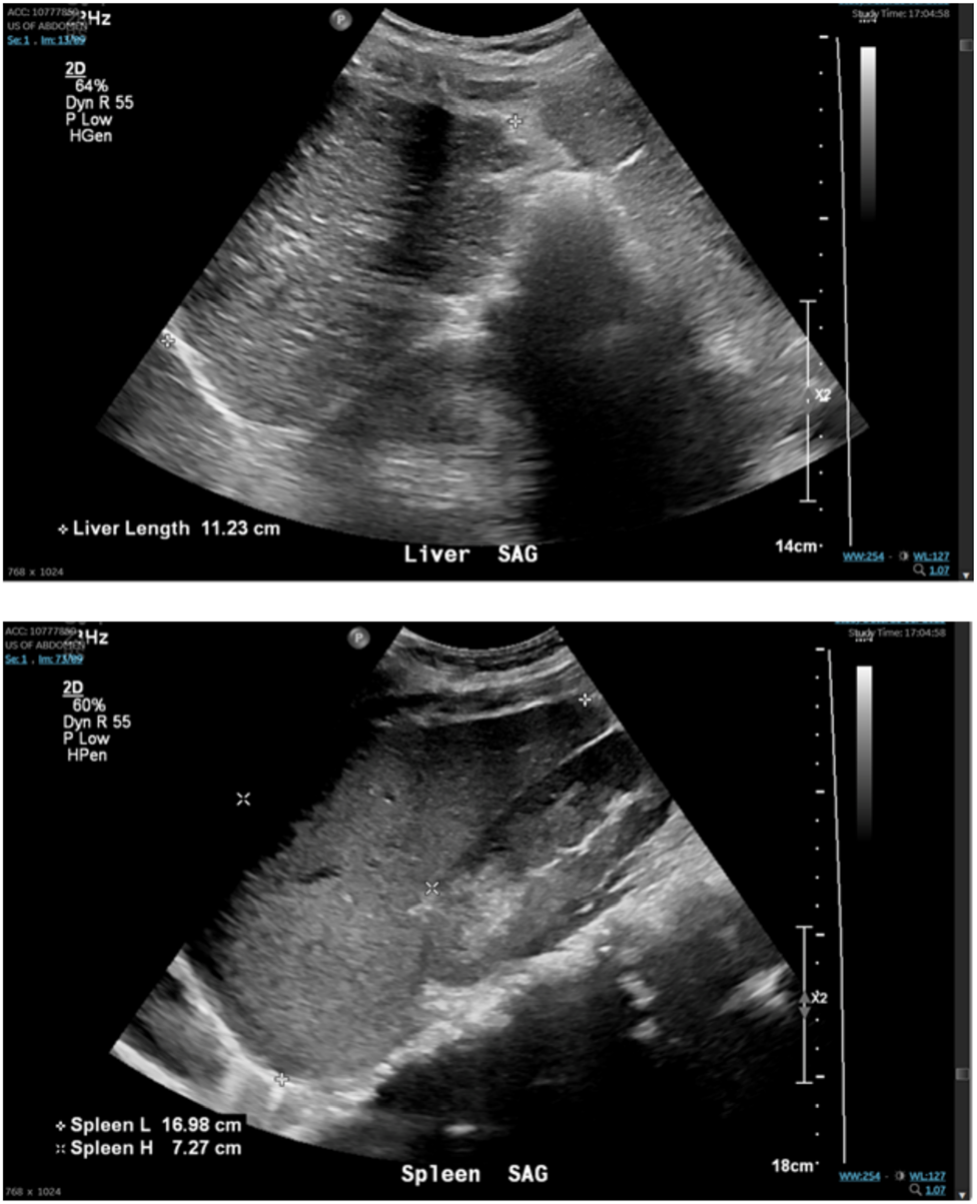
Abdominal ultrasound demonstrating liver cirrhosis and splenomegaly.

A 29-year-old G4P0121 female presented to prenatal care at 7 weeks gestation with preconception diagnosis of AIH with cirrhosis. The patient was incidentally found to be pregnant during workup of new-onset pancytopenia and splenomegaly (see Figure 1). She had a history of spontaneous preterm delivery at 36 weeks complicated by cirrhosis, sepsis, thrombocytopenia, acute kidney injury, and spontaneous bacterial peritonitis (SBP). The current pregnancy was co-managed by Maternal Fetal Medicine, Gastroenterology, Hematology, and Anesthesiology. The patient was maintained on azathioprine, prednisone, and trimethoprim/sulfamethoxazole throughout pregnancy. She developed mild pulmonary hypertension suspected due to right heart disease from AIH.

**Figure 2:**
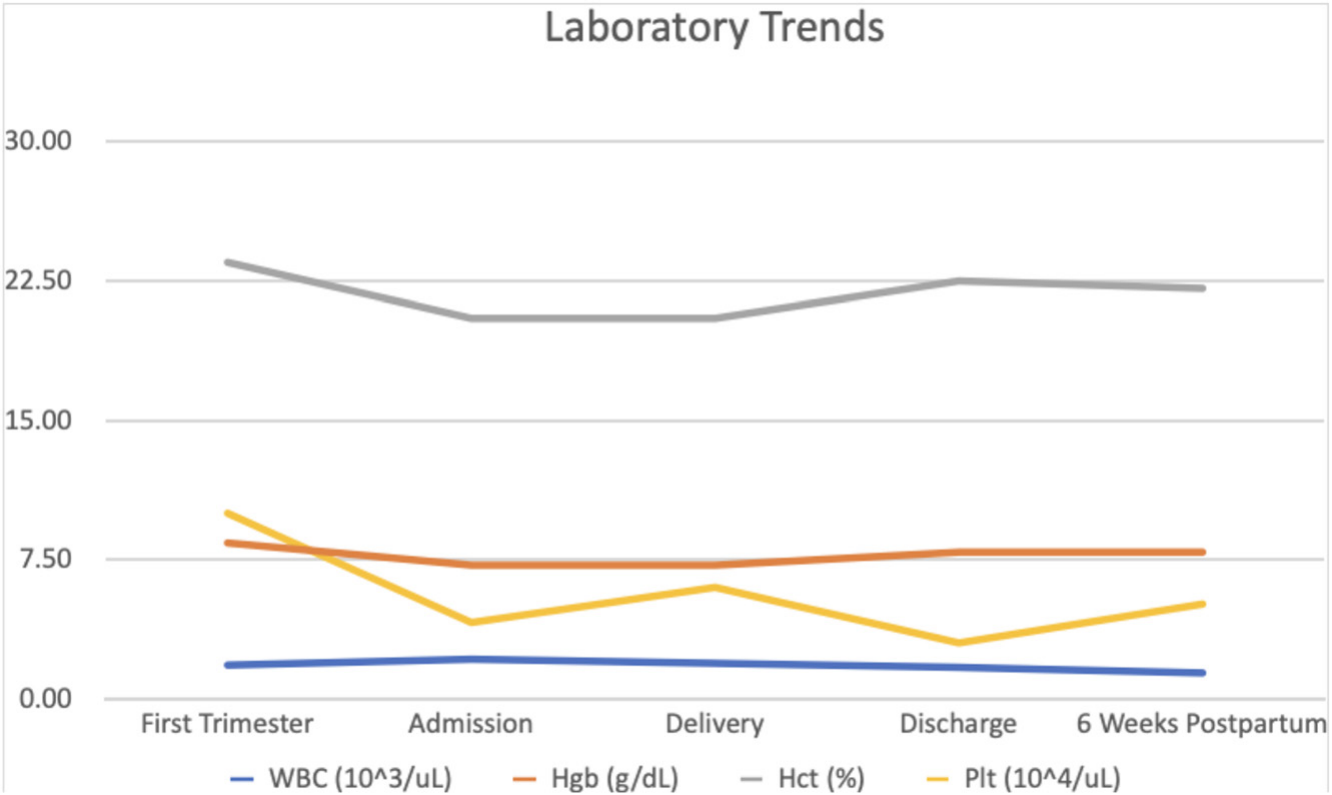
Complete blood count trends during pregnancy course demonstrating severe pancytopenia.

At 32 weeks, she was admitted and evaluated for management of steroid-unresponsive pancytopenia of unclear etiology with severe Coombs-negative anemia unresponsive to packed red blood cell (PRBC) transfusion. Admission labs were as follows: white blood cell count (WBC) 1.60 K/uL, hemoglobin 6.4 g/dL, hematocrit 18.1 %, and platelets 56 K/uL. She received 19U PRBC in transfusions since the start of her pregnancy, but was unable to keep her hemoglobin/hematocrit levels above 8 g/dL and 22.6 % at any given time (see Figure 2).

Fecal occult blood test was positive during admission but EGD/colonoscopy was deferred to postpartum given low suspicion for overt GI bleed or esophageal varices (last EGD in 2022 was negative).

During this admission, delivery was planned for 37 weeks. However, the patient underwent a primary cesarean section for persistent category II fetal heart tracing at 33 weeks gestation and delivered a viable infant. The infant was admitted to the neonatal intensive care unit with mild acute respiratory distress due to apnea of prematurity with APGARs 4 and 8 at 1 and 5 min respectively, cholestasis with direct bilirubinemia, and necrotizing enterocolitis. Given *in utero* exposure to immunosuppressants, neonatal course was complicated by lymphopenia (WBC 2.79 K/uL) on day 1 of life and anemia (hemoglobin 10.6 g/dL and hematocrit 30.3 %) on day 20 of life requiring 1U PRBC transfusion. Workup for comprehensive combined immunodeficiency panel was negative, and infant was discharged home on day 37 of life with resolving lymphocytopenia. Immunology follow-up 1 week later revealed normal lab parameters (WBC 10.02 K/uL, hemoglobin 9.6/28.8 %, platelet 644 K/uL).

For preoperative optimization, the patient was transfused 1U platelets, 4U PRBC packed red blood cells, albumin, and cryoglobulin with additional blood products on call to the operating room. Delivery was complicated by an estimated blood loss of 1 L, but due to her low pre-procedure labs, multiple blood products (3U PRBC, 4U FFP, 1U platelets) as well as tranexamic acid were administered.

Postpartum course was complicated by sepsis secondary to urinary tract infection, and decompensated cirrhosis with hepatic encephalopathy for which she was started on rifaximin and lactulose, and coagulopathy. Bone marrow biopsy for severe pancytopenia showed consistency with hypersplenism without concern for malignancy. Patient has recovered well and was last seen at her 3 month follow-up visit with her cardiologist. Currently, patient continues to be pancytopenic without a return to baseline labs and has traveled abroad with instructions to continue trending her complete blood counts.

## Discussion

The data regarding pregnancy in women with AIH is limited, and few studies exist on pregnancy outcomes in these women [1]. No case reports have documented the management of AIH-induced pancytopenia in pregnancy, although cases have been reported with severe thrombocytopenia [2].

Treatment goals of AIH include improving symptoms, reducing liver inflammation, and preventing disease progression [3, 4]. This starts with glucocorticoid treatment, with azathioprine as maintenance. The overall prognosis of AIH is excellent with a 10-year survival of 90 % [5], however cirrhosis is associated with higher mortality rates [6]. AIH disproportionally affects reproductive-aged females, and AIH in pregnancy is associated with many adverse maternal and fetal outcomes [7, 8].

This case highlights the challenges in management of AIH in pregnancy, particularly the difficulty in balancing the need for immunosuppression with the increased risk of infection that may lead to sequelae such as SBP and sepsis, as well as optimizing delivery planning in the setting of unresponsive pancytopenia. The clinical presentation of AIH can be complex, and demonstrates the need for a multidisciplinary team in the care of patients who have complications.

## Take-home messages

1. AIH can have serious adverse maternal outcomes such as pulmonary hypertension due to right heart strain, severe pancytopenia unresponsive to therapy, SBP, sepsis secondary to need for immunosuppression, and decompensated cirrhosis with hepatic encephalopathy and coagulopathy, which were seen in our patient.
2. Management of AIH requires a multidisciplinary approach for delivery planning as the patient can rapidly decompensate, thus requiring communication with the blood bank, obstetrical anesthesiology, hematology, gastroenterology, cardiology, neonatal intensive care, and the obstetrical team to provide pre-delivery optimization.
3. Consideration of AIH management in pregnancy involves finding a balance between inducing a chronic immunosuppressed state to reduce the complications of cirrhosis and pancytopenia, and mounting an adequate immune response to prevent SBP and sepsis.

## References

[1] Kothadia JP, Shah JM (2023). Autoimmune hepatitis and pregnancy [Internet].

[2] Orgul G, Uyanik Ozkan E, Tolga Celik H, Sinan Beksac M (2017). Autoimmune hepatitis and pregnancy: report of two cases with different maternal outcomes. Clin Exp Hepatol [Internet].

[3] Mack CL, Adams D, Assis DN, Kerkar N, Manns MP, Mayo MJ (2020). Diagnosis and management of autoimmune hepatitis in adults and children: 2019 practice guidance and guidelines from the American Association for the study of liver diseases. Hepatol.

[4] Pape S, Snijders RJALM, Tom OC, Dalekos GN, Manns MP, Hirschfield GM (2022). Systematic review of response criteria and endpoints in autoimmune hepatitis by the International Autoimmune Hepatitis Group. J Hepatol.

[5] Gossard AA, Lindor KD (2012). Autoimmune hepatitis: a review. J Gastroenterol [Internet].

[6] Feld JJ, Dinh H, Arenovich T, Marcus VA, Wanless IR, Heathcote EJ (2005). Autoimmune hepatitis: effect of symptoms and cirrhosis on natural history and outcome. Hepatology.

[7] Schramm C, Herkel J, Ulrich B, Kanzler S, Galle PR, Lohse AW (2006). Pregnancy in autoimmune hepatitis: outcome and risk factors. Am J Gastroenterol.

[8] Heneghan MA (2001). Management and outcome of pregnancy in autoimmune hepatitis. Gut.

